# Functional impact of exercise pulmonary hypertension in patients with borderline resting pulmonary arterial pressure

**DOI:** 10.1177/2045893217709025

**Published:** 2017-06-08

**Authors:** Rudolf K. F. Oliveira, Mariana Faria-Urbina, Bradley A. Maron, Mario Santos, Aaron B. Waxman, David M. Systrom

**Affiliations:** 1 Division of Pulmonary and Critical Care Medicine, Department of Medicine, Brigham and Women’s Hospital and Harvard Medical School, Boston, MA, USA; 2 Heart & Vascular Center, Brigham and Women’s Hospital, Boston, MA, USA; 3 Division of Respiratory Diseases, Department of Medicine, Federal University of São Paulo (UNIFESP), São Paulo, SP, Brazil; 4 Division of Cardiovascular Medicine, Department of Medicine, Brigham and Women’s Hospital and Harvard Medical School, Boston, MA, USA; 5 Veterans Affairs Boston Healthcare System, Boston, MA, USA; 6 Department of Physiology and Cardiothoracic Surgery, Cardiovascular R&D Unit, Faculty of Medicine, University of Porto, Portugal

**Keywords:** pulmonary hypertension, exercise, oxygen uptake, oxygen delivery, pathophysiology

## Abstract

Borderline resting mean pulmonary arterial pressure (mPAP) is associated with adverse outcomes and affects the exercise pulmonary vascular response. However, the pathophysiological mechanisms underlying exertional intolerance in borderline mPAP remain incompletely characterized. In the current study, we sought to evaluate the prevalence and functional impact of exercise pulmonary hypertension (ePH) across a spectrum of resting mPAP’s in consecutive patients with contemporary resting right heart catheterization (RHC) and invasive cardiopulmonary exercise testing. Patients with resting mPAP <25 mmHg and pulmonary arterial wedge pressure ≤15 mmHg (n = 312) were stratified by mPAP < 13, 13–16, 17–20, and 21–24 mmHg. Those with ePH (n = 35) were compared with resting precapillary pulmonary hypertension (rPH; n = 16) and to those with normal hemodynamics (non-PH; n = 224). ePH prevalence was 6%, 8%, and 27% for resting mPAP 13–16, 17–20, and 21–24 mmHg, respectively. Within each of these resting mPAP epochs, ePH negatively impacted exercise capacity compared with non-PH (peak oxygen uptake 70 ± 16% versus 92 ± 19% predicted, *P* < 0.01; 72 ± 13% versus 86 ± 17% predicted, *P* < 0.05; and 64 ± 15% versus 82 ± 19% predicted, *P* < 0.001, respectively). Overall, ePH and rPH had similar functional limitation (peak oxygen uptake 67 ± 15% versus 68 ± 17% predicted, *P* > 0.05) and similar underlying mechanisms of exercise intolerance compared with non-PH (peak oxygen delivery 1868 ± 599 mL/min versus 1756 ± 720 mL/min versus 2482 ± 875 mL/min, respectively; *P* < 0.05), associated with chronotropic incompetence, increased right ventricular afterload and signs of right ventricular/pulmonary vascular uncoupling. In conclusion, ePH is most frequently found in borderline mPAP, reducing exercise capacity in a manner similar to rPH. When borderline mPAP is identified at RHC, evaluation of the pulmonary circulation under the stress of exercise is warranted.

## Introduction

Pulmonary hypertension (PH) is currently defined by resting mean pulmonary arterial pressure (mPAP) ≥25 mmHg during a supine right heart catheterization (RHC).^1^ However, the normal resting mPAP value is known to be of 14 ± 3 mmHg and the upper limit of normal is approximately 20 mmHg.^2^ Consequently, a substantial number of patients have abnormal resting mPAP values, though within the non-diagnostic PH range.

There is growing evidence that borderline mPAP (21–24 mmHg) is associated with worse long-term outcomes. Recently, Maron et al. showed in a large heterogeneous population that resting mPAP 19–24 mmHg is associated with increased risk for hospitalization and mortality.^3^ Similarly, Kovacs et al. reported resting mPAP 21–24 mmHg to be associated with decreased exercise capacity and decreased survival.^4^ Furthermore, in patients with known risk factors for pulmonary vascular disease such as systemic sclerosis, several studies have demonstrated that borderline mPAP might be a unique clinical phenotype with associated worse prognosis.^5–7^

Similar to resting borderline mPAP, recent evidence suggests pre-capillary PH diagnosed during exercise (ePH) is a clinically relevant condition that is associated with symptoms, impacts exercise capacity and outcomes, and may progress to resting PH and might reflect an early (and likely more treatable) stage of established PH.^8–15^ Additionally, recent work from Lau et al. suggests ePH occurrence is high in borderline resting mPAP,^16^ pointing to the possible close association between these two conditions. However, the pathophysiologic link between borderline mPAP and ePH and the resultant functional impact of ePH in borderline mPAP remains incompletely characterized.

In the current study, we sought to evaluate the prevalence and functional implications of ePH across a spectrum of resting mPAP currently considered to be normal, including borderline mPAP, and to contrast ePH pathophysiological implications to those of resting precapillary pulmonary hypertension (rPH). We hypothesized ePH occurrence increases as a function of higher resting mPAP values, impacting exercise capacity in a manner similar to rPH.

## Methods

We analyzed retrospectively 723 consecutive patients referred to the Brigham and Women’s Hospital Dyspnea Center over a 5.5-year period (from January 2012 until June 2016) with suspected PH who underwent resting supine RHC followed by an upright symptom-limited invasive cardiopulmonary exercise testing (iCPET) as part of their clinically indicated evaluation for unexplained exertional intolerance.^17^ The study protocol was approved by the Partners Human Research Committee (2011P000272).

**Fig. 1.**
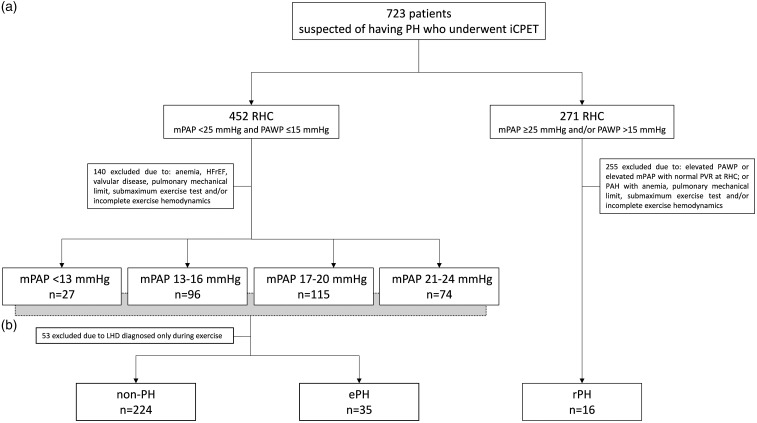
Study flow diagram. PH, pulmonary hypertension; iCPET, invasive cardiopulmonary exercise testing; RHC, right heart catheterization; mPAP, mean pulmonary arterial pressure; PAWP, pulmonary arterial wedge pressure; HFrEF, heart failure with reduced ejection fraction; PVR, pulmonary vascular resistance; LHD, left heart disease; non-PH, normal resting/exercise pulmonary hemodynamics; ePH, exercise pulmonary hypertension; rPH, resting precapillary pulmonary hypertension.

According to resting supine RHC, patients were classified in two groups: those with mPAP < 25 mmHg and pulmonary arterial wedge pressure (PAWP) ≤15 mmHg; and those with mPAP ≥ 25 mmHg and/or PAWP > 15 mmHg (Fig. 1). For the primary analysis, and in order to evaluate the prevalence and functional implications of ePH, as a function of increasing resting mPAP at RHC, patients with mPAP < 25 mmHg and PAWP ≤ 15 mmHg were divided in four subgroups according to regular increasing resting mPAP intervals (<13 mmHg, 13–16 mmHg, 17–20 mmHg, and 21–24 mmHg) (Fig. 1a). For the secondary analysis, and to evaluate ePH pathophysiological implications in relation to patients with resting established disease, ePH iCPET results were contrasted to those with rPH and those with normal resting/exercise pulmonary hemodynamics (non-PH) (Fig. 1b).

Exclusion criteria included: (1) anemia defined by hemoglobin concentration <10 g.dL^−1^; (2) left heart disease (LHD) defined by moderate/severe mitral and/or aortic valvular disease or left ventricular ejection fraction <0.5 at resting echocardiography, or postcapillary PH identified by mPAP ≥ 25 mmHg and PAWP > 15 mmHg at resting RHC or PAWP ≤ 15 mmHg at rest but abnormally elevated during exercise associated with a normal peak pulmonary vascular resistance (PVR) for the patient’s age (i.e. peak PAWP > 19 mmHg and peak PVR ≤ 1.35 WU for patients aged ≤50 years or PAWP > 17 mmHg and peak PVR ≤ 2.10 WU for patients aged >50 years);^18^ (3) pulmonary mechanical limitation to exercise defined by ventilatory reserve at the anaerobic threshold ≥0.70;^19^ (4) submaximal cardiopulmonary exercise testing defined by peak respiratory exchange ratio (RER) <1.05 and peak heart rate <85% predicted and peak mixed-venous partial pressure of oxygen <27 mmHg;^20^ and (5) incomplete exercise hemodynamics.

Baseline demographics, anthropometrics, co-morbidities, and contemporary pulmonary function testing and resting echocardiography were reviewed. A relevant lung disease was defined by the forced expiratory volume in the first second divided by forced vital capacity (FEV_1_/FVC) <70% predicted associated with FEV_1_ <60% predicted, or a radiologic diagnosis of lung fibrosis associated with a FVC <70% predicted.^21^

### Pulmonary hypertension definitions

ePH was defined by age-specific exercise pulmonary hemodynamic criteria for maximum upright exercise as follows: (1) peak mPAP > 30 mmHg and peak PVR > 1.34 WU for patients aged ≤ 50 years; or (2) peak mPAP > 33 mmHg and peak PVR > 2.10 WU for patients aged > 50 years.^18^ rPH was defined by resting supine RHC as mPAP ≥ 25 mmHg and PAWP ≤ 15 mmHg and PVR > 3 WU.^22^

### Hemodynamic measurements

Our RHC and iCPET methods have been described in previous studies^14,17,18^ and is its technical aspects have been reported in detail elsewhere.^23^ Briefly, RHC was performed in the supine position with a pulmonary arterial catheter (Edwards Lifesciences, Irvine, CA, USA) inserted percutaneously via the internal jugular vein and a concurrent catheter placed via the radial artery following a negative Allen’s test. Cardiac output (CO) at resting RHC was calculated using Fick’s method and an estimated oxygen consumption (VO_2_).

With patients breathing room air, a symptom-limited incremental CPET was next performed using an upright cycle ergometer and a breath-by-breath metabolic cart (ULTIMA CPX; Medical Graphics Corporation, St Paul, MN, USA). Pulmonary and systemic hemodynamics were continuously and simultaneously monitored during exercise (Xper Cardio Physiomonitoring System; Philips, Melborne, FL, USA). Pulmonary pressures were recorded at the end of a passive exhalation;^23^ when respirophasic changes persisted, an electronic average over three respiratory cycles was used.^24^ Arterial and mixed venous blood gases and pH were collected during each minute of exercise, and arterial-mixed venous oxygen content difference (Ca-vO_2_) was calculated. By the Fick principle and using a simultaneously measured VO_2_, minute per minute CO was than obtained during exercise. Oxygen delivery (DO_2_) was calculated multiplying CO by the arterial oxygen content (CaO_2_).

Total pulmonary vascular resistance (TPR) and PVR were calculated by mPAP/CO and mPAP–PAWP/CO, respectively, and expressed as Wood units (WU). Pulmonary vascular compliance (PVC) was calculated by: stroke volume/systolic–diastolic PAP and expressed as mL/mmHg. Right ventricular stroke work index (RVSWI) was calculated by: (1.25 mPAP–right atrial pressure × stroke volume index × 0.0136)^25–27^ and expressed as g/m/m^2^.

### Statistical analysis

Unless otherwise stated, values are presented as mean and standard deviation or median and interquartile range. Group comparisons were performed using Chi-squared, Fisher’s exact tests, t-test, or Mann–Whitney U-test as appropriate. One-way ANOVA with Tukey’s post hoc analysis was used when comparing more than two groups. Receiver operating characteristic (ROC) curve analyses were derived for resting pulmonary hemodynamics while accounting for the presence or absence of ePH. *P* < 0.05 was considered significant. The statistical analyses were performed using SPSS software, version 19 (IBM Company, Armonk, NY, USA).

## Results

Of the 723 iCPET reports analyzed, 271 (37%) had resting supine mPAP ≥ 25 mmHg and/or PAWP > 15 mmHg and 452 (63%) had mPAP < 25 mmHg and PAWP ≤ 15 mmHg at RHC (Fig. 1). For the primary analysis (Fig. 1a), 140 patients with mPAP < 25 mmHg and PAWP ≤ 15 mmHg were excluded due to anemia, echocardiographic abnormalities, pulmonary mechanical limitation to exercise, submaximum exercise test, and/or incomplete data. For the secondary analysis (Fig. 1b), 255 patients with mPAP ≥ 25 mmHg and/or PAWP > 15 mmHg were excluded based on RHC (elevated PAWP or elevated mPAP with normal PVR) or iCPET data (rPH with anemia, pulmonary limit, submaximum exercise test, and/or incomplete exercise hemodynamics), and 53 patients with mPAP < 25 mmHg and PAWP ≤ 15 mmHg were excluded due to LHD diagnosed only during exercise.

### Resting mPAP < 13 versus 13–16 versus 17–20 versus 21–24 mmHg

In total, 312 patients with mPAP < 25 mmHg and PAWP ≤ 15 mmHg were analyzed. Twenty-seven patients had mPAP < 13 mmHg, 96 mPAP 13–16 mmHg, 115 mPAP 17–20 mmHg, and 74 mPAP 21–24 mmHg (Fig. 1a). Their resting mPAP distribution is presented in the online supplementary material (Fig. S1).

Age, body mass index (BMI), and the presence of co-morbidities increased as a function of higher resting mPAP values and a mPAP within the borderline range (21–24 mmHg) was significantly associated with older age and elevated BMI. At RHC, there was progressively higher PVR and TPR and lower PVC as a function of higher mPAPs. Their baseline characteristics and RHC data are summarized in Table 1.

**Table 1.** Baseline characteristics according to the resting mean pulmonary arterial pressure (mPAP) at right heart catheterization.

	mPAP < 13 mmHg	mPAP 13–16 mmHg	mPAP 17–20 mmHg	mPAP 21–24 mmHg
*Participants*	27	96	115	74
Age (years)	49 ± 11	51 ± 16	56 ± 16	62 ± 13*^†‡^
Women (n (%))	17 (63)	70 (73)	65 (67)	39 (53)
BMI (kg/m^2^)	26.3 ± 5.8	26.6 ± 6.4	29.1 ± 6.5*	30.1 ± 6.2*^†^
Hemoglobin (g/dL)	14.3 ± 1.4	14.0 ± 1.5	14.2 ± 1.6	13.7 ± 1.5
*Co-morbidities (n (%))*				
None	17 (63)	48 (50)	41 (36)	19 (26)
Hypertension	4 (15)	28 (29)	50 (43)	38 (51)
Connective tissue disease	2 (7)	6 (6)	11 (10)	12 (16)
Diabetes mellitus	1 (4)	3 (3)	13 (11)	9 (12)
Lung disease	0	2 (2)	5 (4)	6 (8)
Smokers	0	3 (3)	4 (3)	1 (1)
History of pulmonary embolism	0	6 (6)	8 (7)	7 (9)
*Medications (n (%))*				
Diuretics	4 (15)	10 (10)	23 (20)	16 (22)
ACE inhibitor or ARB	6 (22)	26 (27)	44 (38)	29 (39)
Beta-adrenergic receptor blocker	2 (7)	11 (11)	26 (23)	19 (26)
Calcium channel blocker	2 (7)	8 (8)	13 (11)	12 (16)
*Pulmonary function testing*				
FEV_1_ (% predicted)	97 ± 13	94 ± 16	87 ± 19*^†^	82 ± 18*^†^
FVC (% predicted)	98 ± 13	94 ± 17	87 ± 19*^†^	83 ± 17*^†^
FEV_1_/FVC (% predicted)	99 ± 5	100 ± 8	99 ± 10	98 ± 12
*Echocardiography*				
LA AP diameter (mm)	35 ± 4	35 ± 5	36 ± 5	38 ± 6*^†^
LVEF (%)	63 ± 6	62 ± 4	62 ± 4	62 ± 5
TRV (m/s)	2.2 ± 0.3	2.3 ± 0.3	2.4 ± 0.4*	2.5 ± 0.3*^†^
Estimated sPAP (mmHg)	22 ± 9	23 ± 5	27 ± 7*	28 ± 7*^†^
*Right heart catheterization*				
RA (mmHg)	4 ± 2	5 ± 2	7 ± 2*^†^	7 ± 3*^†^
mPAP (mmHg)	11 ± 1	15 ± 1*	18 ± 1*^†^	22 ± 1*^†‡^
PAWP (mmHg)	6 ± 2	9 ± 2*	11 ± 2*^†^	13 ± 3*^†‡^
TPG (mmHg)	5 ± 2	6 ± 2	7 ± 3*^†^	9 ± 3*^†‡^
CO (L/min)	4.8 ± 1.0	5.4 ± 1.1	5.4 ± 1.1	5.5 ± 1.3
CI (L/min/m^2^)	2.7 ± 0.6	2.9 ± 0.6	2.8 ± 0.5	2.8 ± 0.6
TPR (WU)	2.5 ± 0.7	2.9 ± 0.6	3.5 ± 0.7*^†^	4.3 ± 1.2*^†‡^
PVR (WU)	1.0 ± 0.4	1.1 ± 0.4	1.4 ± 0.6*^†^	1.8 ± 0.7*^†‡^
PVC (mL/mmHg)	7.3 ± 2.4	6.5 ± 2.2	5.8 ± 1.9*	4.7 ± 1.9*^†^

Data are presented as n, n (%), or mean ± standard deviation.

* *P* < 0.05 compared with mPAP < 13 mmHg.

† *P* < 0.05 compared with mPAP 13–16 mmHg.

‡ *P* < 0.05 compared with mPAP 17–20 mmHg.

BMI, body mass index; ACE, angiotensin-converting- enzyme; ARB, angiotensin II receptor antagonist; FEV_1_, forced expiratory volume in 1 s; FVC, forced vital capacity; LA AP, left atrium anteroposterior; LVEF, left ventricular ejection fraction; TRV, tricuspid regurgitant jet velocity; sPAP, systolic pulmonary arterial pressure; RAP, right atrial pressure; mPAP, mean pulmonary arterial pressure; PAWP, pulmonary arterial wedge pressure; TPG, transpulmonary gradient; CO, cardiac output; CI, cardiac index; TPR, total pulmonary vascular resistance; PVR, pulmonary vascular resistance; PVC, pulmonary vascular compliance.

During iCPET, higher resting mPAP values were associated with decreased exercise capacity, as measured by peak VO_2_ (Table 2). Also, decreased peak CaO_2_ and decreased peak DO_2_ were observed in borderline mPAP. Exercise hemodynamics additionally revealed decreased peak CO and increased peak right atrial pressure, peak TPR, and peak PVR in patients with resting borderline mPAP (Table 2).

**Table 2.** Upright invasive cardiopulmonary exercise data according to the resting mean pulmonary arterial pressure (mPAP) at right heart catheterization.

	mPAP < 13 mmHg	mPAP 13–16 mmHg	mPAP 17–20 mmHg	mPAP 21–24 mmHg
*Participants*	27	96	115	74
Maximum work rate (W)	137 ± 50	134 ± 54	123 ± 49	102 ± 42*^†‡^
Peak VO_2_ (% predicted)	90 ± 21	91 ± 22	83 ± 18*^†^	75 ± 19*^†‡^
Peak VO_2_ (mL/kg/min)	23.6 ± 8.1	22.5 ± 8.5	19.2 ± 6.4*^†^	15.7 ± 5.3*^†‡^
VO_2_ at AT (% VO_2MAX_ predicted)	49 ± 12	51 ± 17	45 ± 10^†^	43 ± 13^†^
Peak heart rate (bpm)	151 ± 18	151 ± 25	142 ± 26^†^	129 ± 27*^†‡^
Peak heart rate (% predicted)	88 ± 11	89 ± 11	86 ± 12	80 ± 14*^†‡^
Peak RER	1.19 ± 0.12	1.14 ± 0.12	1.14 ± 0.13	1.12 ± 0.11
Peak VE/MVV	64 ± 16	60 ± 16	66 ± 21	64 ± 17
VE/VCO_2_ slope	32 ± 8	31 ± 6	32 ± 8	36 ± 10^†‡^
Peak P_A − a_O_2_ (mmHg)	11 ± 11	17 ± 12	18 ± 19	28 ± 19*^†‡^
Peak SaO_2_ (%)	98 ± 1	97 ± 2	96 ± 7	95 ± 4
Peak CaO_2_ (mL/dL)	19.5 ± 1.9	19.1 ± 2.4	19.5 ± 2.3	18.3 ± 2.2^‡^
Peak Ca-vO_2_ (mL/dL)	13.2 ± 1.8	12.6 ± 2.0	12.7 ± 2.0	12.2 ± 2.0
Peak DO_2_ (mL/min)	2535 ± 884	2541 ± 930	2490 ± 871	2012 ± 707*^†‡^
Peak DO_2_ (mL/kg/min)	34.7 ± 11.4	34.2 ± 12.4	29.8 ± 9.8^†^	23.5 ± 9.8*^†‡^
*Exercise hemodynamics*				
Peak RAP (mmHg	5 ± 4	6 ± 4	7 ± 5	9 ± 5*^†‡^
Peak mPAP (mmHg)	23 ± 6	26 ± 7	29 ± 8*^†^	34 ± 8*^†‡^
Peak PAWP (mmHg)	10 ± 4	12 ± 5	14 ± 6*	16 ± 6*^†^
Peak TPG (mmHg)	13 ± 4	14 ± 5	16 ± 6	19 ± 7*^†‡^
Peak CO (L/min)	12.8 ± 3.7	13.1 ± 3.9	12.7 ± 3.9	10.9 ± 3.3^†‡^
Peak CI (L/min/m^2^)	7.0 ± 1.8	7.0 ± 1.9	6.4 ± 1.7^†^	5.5 ± 1.5*^†‡^
Peak SV (mL)	85 ± 23	88 ± 25	90 ± 24	86 ± 20
Peak SVI (mL/m^2^)	47 ± 12	47 ± 11	46 ± 10	43 ± 7^†^
Peak TPR (WU)	1.9 ± 0.5	2.2 ± 0.8	2.5 ± 0.9*	3.5 ± 1.5*^†‡^
Peak PVR (WU)	1.1 ± 0.4	1.2 ± 0.6	1.3 ± 0.6	1.8 ± 0.9*^†‡^
Peak PVC (mL/mmHg)	3.6 ± 1.4	3.6 ± 1.6	3.3 ± 1.4	3.1 ± 1.2
Peak RVSWI (g/m/m^2^)	15.4 ± 6.6	17.4 ± 6.7	18.6 ± 6.8	19.8 ± 6.6*

Data are presented as n or mean ± standard deviation.

* *P* < 0.05 compared with mPAP < 13 mmHg.

† *P* < 0.05 compared with mPAP 13–16 mmHg.

‡ *P* < 0.05 compared with mPAP 17–20 mmHg.

VO_2_, oxygen uptake; VO_2MAX_, maximal oxygen uptake; AT, anaerobic threshold; RER, respiratory exchange ratio; VE/MVV, ventilatory reserve; VE/VCO_2_, ventilatory equivalent for carbon dioxide; P_A−a_O_2_, alveolar–arterial oxygen tension difference; SaO_2_, arterial oxygen saturation; CaO_2_, arterial oxygen content; Ca-vO_2_, arterial–mixed venous oxygen content difference; DO_2_, oxygen delivery; RAP, right atrial pressure; mPAP, mean pulmonary arterial pressure; PAWP, pulmonary arterial wedge pressure; TPG, transpulmonary gradient; CO, cardiac output; CI, cardiac index; SV, stroke volume; SVI, stroke volume index; TPR, total pulmonary vascular resistance; PVR, pulmonary vascular resistance; PVC, pulmonary vascular compliance; RVSWI, right ventricular stroke work index.

**Fig. 2.**
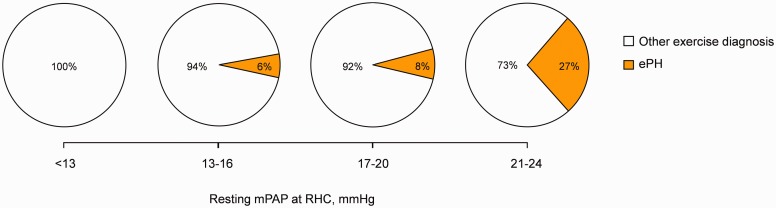
Prevalence of exercise pulmonary hypertension (ePH) according to the resting mean pulmonary arterial pressure (mPAP) at right heart catheterization (RHC).

Based on the aforementioned exercise hemodynamic criteria, ePH occurred in 6% (6 out of 96) of patients with mPAP 13–16 mmHg, 8% (9 out of 115) of patients with mPAP 17–20 mmHg and 27% (20 out of 74) of those with mPAP 21–24 mmHg (Fig. 2).

### ePH versus rPH versus non-PH

In total, 35 ePH, 16 rPH, and 224 non-PH patients were analyzed (Fig. 1b). Compared with non-PH, ePH had higher resting mPAP (20 ± 3 mmHg versus 17 ± 3 mmHg, *P* < 0.05) and PVR (2.1 ± 0.8 WU versus 1.3 ± 0.5 WU, *P* < 0.05) and lower PVC (4.3 ± 1.6 mL/mmHg versus 6.2 ± 2.1 mL/mmHg, *P* < 0.05) at RHC. rPH had resting mPAP of 36 ± 11 mmHg, PAWP 10 ± 3 mmHg, TPG 27 ± 10 mmHg, PVR 5.7 ± 2.8 WU at RHC. rPH and ePH tended to be older compared with non-PH (62 ± 11 versus 59 ± 16 versus 53 ± 16, *P* = 0.02, respectively) and had more co-morbidities such as systemic hypertension, connective tissue disease, diabetes mellitus, and history of pulmonary embolism. Detailed baseline characteristics and RHC data for rPH, ePH, and non-PH are presented in the online supplementary material (Table S1).

**Fig. 3.**
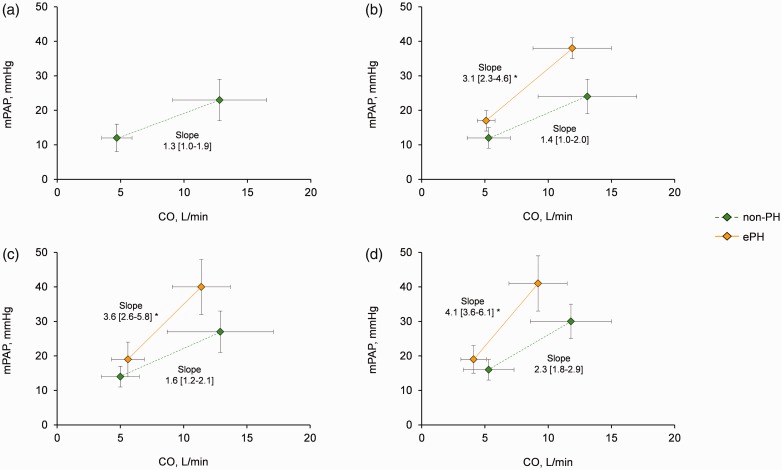
Mean pulmonary arterial pressure to cardiac output (mPAP/CO) slope from rest upright to peak upright according to the right heart catheterization mPAP. mPAP < 13 mmHg (a), mPAP 13–16 mmHg (b), mPAP 17–20 mmHg (c), and mPAP 21–24 mmHg (d). Slope values are presented as median [interquartile range]. non-PH, normal resting/exercise pulmonary hemodynamics; ePH, exercise pulmonary hypertension. *P < 0.05 compared with non-PH.

**Fig. 4.**
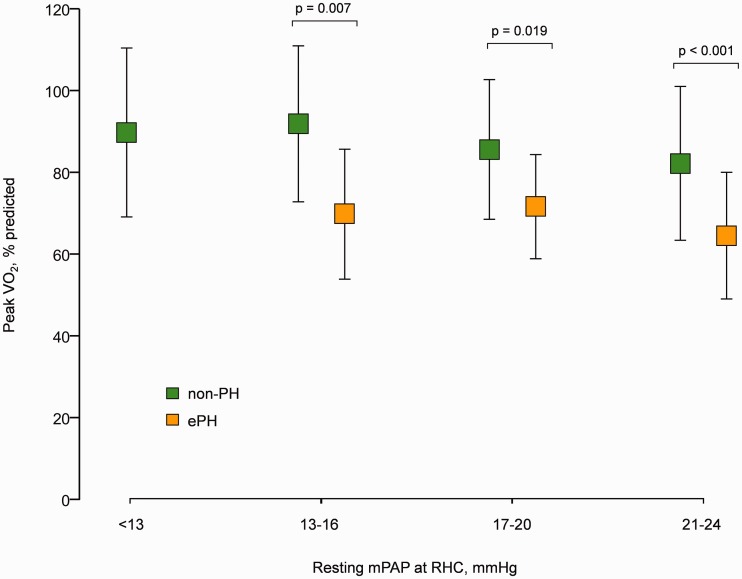
Peak oxygen uptake (VO_2_) across different resting mean pulmonary arterial pressure (mPAP) values at right heart catheterization. Data represent the mean ± SD (error bars). non-PH, normal resting/exercise pulmonary hemodynamics; ePH, exercise pulmonary hypertension.

Patients with ePH had elevated mPAP/CO slopes compared with non-PH across the different mPAP ranges (Fig. 3), and within each resting mPAP epoch, the development of ePH was associated with a significantly reduced peak VO_2_ as a % predicted compared with non-PH (Fig. 4).

In the aggregate, ePH and rPH had similarly reduced exercise capacity compared with non-PH as shown by their reduced peak VO_2_, which in turn was associated with decreased peak CaO_2_, peak CO, and peak DO_2_, and indices of decreased right ventricular function and increased right ventricular and pulmonary vascular load (Table 3). ePH and rPH also showed chronotropic incompetence at peak exercise (Table 3). In ePH, chronotropic incompetence occurred predominately in patients aged > 50 years (Table S2) and irrespective of the use of beta-adrenergic receptor blockers (Table S3).

**Table 3.** Functional and pathophysiological determinants of exercise pulmonary hypertension (ePH) and resting precapillary pulmonary hypertension (rPH) during upright invasive cardiopulmonary exercise testing.

	Non-PH	ePH	rPH
*Participants*	224	35	16
*Exercise capacity*			
Maximum work rate (W)	129 ± 49	99 ± 43*	77 ± 49*
Peak VO_2_ (% predicted)	88 ± 19	67 ± 15*	68 ± 17*
Peak VO_2_ (mL/kg/min)	21.1 ± 7.2	14.7 ± 4.4*	14.3 ± 5.8*
VO_2_ at AT (% VO_2MAX_ predicted)	48 ± 12	40 ± 11*	39 ± 11*
*Determinants of exercise capacity*			
Peak CaO_2_ (mL/dL)	19.3 ± 2.2	18.1 ± 2.4*	16.7 ± 2.0*
Peak CvO_2_ (mL/dL)	6.6 ± 1.7	6.2 ± 2.1	6.3 ± 1.8
Peak Ca-vO_2_ (mL/dL)	12.7 ± 1.9	12.0 ± 2.5	10.4 ± 1.8*^†^
Peak CO (L/min)	12.8 ± 3.9	10.2 ± 2.7*	10.3 ± 3.4*
Peak CI (L/min/m^2^)	6.7 ± 1.9	5.3 ± 1.2*	5.6 ± 1.7*
Peak SV (mL)	88 ± 24	82 ± 14	79 ± 22
Peak SVI (mL/m^2^)	46 ± 10	41 ± 6*	43 ± 10
Peak heart rate (bpm)	147 ± 25	128 ± 28*	131 ± 23*
Peak heart rate (% predicted)	88 ± 11	78 ± 13*	82 ± 11
Peak DO_2_ (mL/min)	2482 ± 875	1868 ± 599*	1756 ± 720*
Peak DO_2_ (mL/kg/min)	32.3 ± 11.1	22.1 ± 6.7*	23.1 ± 10.2*
*Pulmonary pressures*			
Peak mPAP (mmHg)	26 ± 6	40 ± 7*	58 ± 11*^†^
Peak PAWP (mmHg)	11 ± 4	15 ± 6*	15 ± 7*
Peak TPG (mmHg)	15 ± 5	25 ± 6*	43 ± 10*^†^
*Indices of right ventricular and pulmonary vascular load*			
Peak RAP (mmHg)	5 ± 4	10 ± 6*	9 ± 5*
Peak TPR (WU)	2.2 ± 0.7	4.3 ± 1.6*	6.1 ± 2.3*^†^
Peak PVR (WU)	1.3 ± 0.6	2.5 ± 0.9*	4.6 ± 1.8*^†^
Peak PVC (mL/mmHg)	3.5 ± 1.4	2.6 ± 1.2*	1.6 ± 0.7*
Peak RVSWI (g/m/m^2^)	16.9 ± 6.4	22.7 ± 5.6*	36.7 ± 10.9*^†^

Data are presented as n or mean ± standard deviation.

* *P* < 0.05 compared with non-PH.

† *P* < 0.05 comparing rPH vs. ePH.

VO_2_, oxygen uptake; VO_2MAX_, maximal oxygen uptake; AT, anaerobic threshold; CaO_2_, arterial oxygen content; CvO_2_, mixed-venous oxygen content; Ca-vO_2_, arterial–mixed venous oxygen content difference; CO, cardiac output; CI, cardiac index; SV, stroke volume; SVI, stroke volume index; DO_2_, oxygen delivery; mPAP, mean pulmonary arterial pressure; PAWP, pulmonary arterial wedge pressure; TPG, transpulmonary gradient; RAP, right atrial pressure; TPR, total pulmonary vascular resistance; PVR, pulmonary vascular resistance; PVC, pulmonary vascular compliance; RVSWI, right ventricular stroke work index.

rPH additionally had a reduced peak Ca-vO_2_ (Table 3). Peak alveolar-arterial oxygen tension difference was elevated in rPH and ePH compared with non-PH (64 ± 14 mmHg versus 35 ± 20 mmHg versus 17 ± 16 mmHg, respectively; *P* < 0.05). Arterial oxygen saturation was reduced in rPH and ePH compared with non-PH (85 ± 6% versus 93 ± 4% versus 97 ± 5%, respectively; *P* < 0.05).

**Fig. 5.**
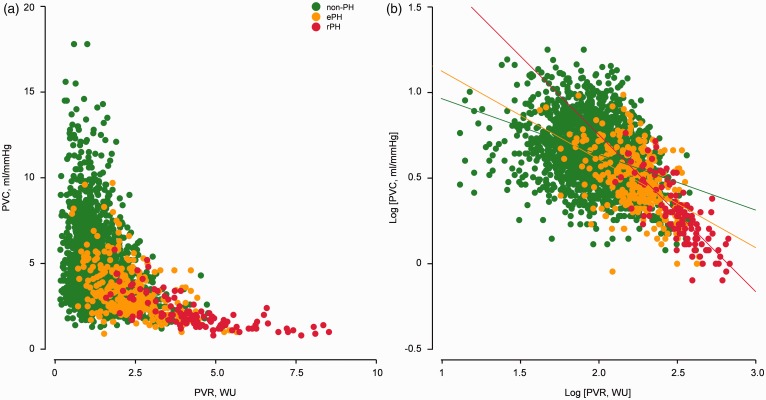
Minute per minute relationship between pulmonary vascular resistance (PVR) and pulmonary vascular compliance (PVC) from rest upright to peak exercise, which included rest, freewheeling and minute per minute incremental PVR and PVC until symptom-limited peak exercise (a). Minute per minute Log [PVR] – log [PVC] plot (b). n = 2129 individual data points obtained from 224 non-PH patients, n = 294 from 35 ePH, and n = 115 from 16 rPH patients. non-PH, normal resting/exercise pulmonary hemodynamics; ePH, exercise pulmonary hypertension; rPH, resting precapillary pulmonary hypertension.

The analysis of minute per minute PVR versus PVC relationship during exercise (n = 2129 individual data points obtained from the 224 non-PH patients, n = 294 from the 35 ePH and n = 115 from the 16 rPH) revealed that ePH pulmonary hemodynamic response to exercise was intermediate between non-PH and rPH (Fig. 5a). Additionally, by the logarithmic transformation of PVR and PVC (Fig. 5b), a progressive downward-leftward change of PVR versus PVC relation was observed for ePH compared with non-PH during exercise, reflecting the dynamic pulmonary vascular responses that led ePH to be an intermediate pulmonary hemodynamic stage between normality and established disease.

### RHC ePH predictors

By ROC analysis, resting supine mPAP, PVR, and PVC provided prediction information about the presence or absence of ePH (AUC = 0.76, 95% CI = 0.68–0.85, *P* < 0.01; AUC = 0.78, 95% CI = 0.69–0.88, *P* < 0.01; and AUC = 0.76, 95% CI = 0.67–0.84, *P* < 0.01, respectively). Their optimal cutoff values to separate ePH from non-PH were 19 mmHg for resting mPAP, 1.6 WU for resting PVR, and 4.6 mL/mmHg for resting PVC. Of the ePH patients, 83% had a mPAP ≥ 19 mmHg and/or a PVR ≥ 1.6 WU at resting supine RHC.

## Discussion

In the present study, we demonstrate that the prevalence of ePH increases as a function of higher resting mPAP values and is found in 27% of the patients with borderline mPAP (21–24 mmHg). When present, ePH substantively affects exercise capacity regardless of the resting mPAP range and the functional impact of ePH is similar than that observed in rPH. ePH and rPH have shared mechanisms of exercise intolerance, suggesting that ePH represents an early pathophysiological stage of rPH. The current findings indicate that the identification of borderline mPAP at RHC should encourage the evaluation of the pulmonary circulation under the stress of exercise to uncover clinically relevant early pulmonary vascular disease.

### Prevalence of ePH in borderline mPAP

Our results reveal that ePH prevalence is elevated in borderline mPAP (Fig. 2), but lower than that previously reported.^16^ Lau et al.^16^ recently described a 65% prevalence of pre-capillary PH during exercise for mPAP 21–24 mmHg;^16^ however, the aforementioned study evaluated patients from a PH Referral Center with an elevated prevalence of PH risk factors.^16^ Conversely, the present study evaluated patients from a Dyspnea Center, which encompass a heterogenous patient population with unexplained exertional intolerance. To our knowledge, the current study is the largest to date to evaluate directly measured invasive exercise hemodynamics as a function of increasing resting mPAP values in a general population undergoing dyspnea investigation and therefore, might more accurately reflect ePH real-life prevalence across different resting mPAP ranges. Despite differences in ePH prevalence, our findings and those of Lau et al.^16^ agree that increasing resting mPAP values are associated with increased ePH occurrence, reinforcing the notion that borderline mPAP should be considered a population at risk for ePH development.

We additionally observed that increasing resting mPAP values were associated with increasing age, BMI, and co-morbidities rate (Table 1), which were followed by progressively abnormal exercise pulmonary hemodynamics (Table 2). These findings are in accord with those of others^3,4^ and likely reflect the association between the presence of co-morbidities and PH risk factors and the development of abnormal pulmonary vascular responses during exercise.

### Functional impact of ePH in borderline mPAP

A key finding of the current study is that regardless of the resting mPAP range, the development of ePH is associated with a reduced exercise capacity (Fig. 4). This finding highlights the additive value of exercise hemodynamics to detect early disease. Additionally, using simultaneously measured maximum incremental VO_2_ (and its indirect determinants) and invasive pulmonary hemodynamics, we demonstrate for the first time that ePH and rPH have similar mechanisms underlying exercise intolerance, specifically, impaired DO_2_ and indices of increased right ventricular and pulmonary vascular load and chronotropic incompetence (Table 3).

The decreased peak DO_2_ was associated with concurrent reduction of peak CaO_2._ and peak CO for both ePH and rPH. While the reduced peak CaO_2_ reflects the known impact of pulmonary vascular disease on the oxygen diffusion through the vascular bed,^28^ the decreased peak CO is likely a result of increased right ventricular afterload (seen by abnormal peak PVR and peak PVC) and right ventricular/pulmonary vascular uncoupling (suggested by the elevated peak right atrial pressure and RVSWI) in both ePH and rPH (Table 3).

Right ventricular maladaptation to increased pulmonary vascular load is a known phenomenon in pulmonary arterial hypertention.^29–31^ In the current study, we found signs of dynamic (only during exercise) right ventricular/pulmonary vascular uncoupling in ePH associated with elevated right heart afterload and right ventricular work, in a similar manner to that found for rPH (Table 3). Ventriculoarterial uncoupling is associated with worse prognosis in established PH;^32^ therefore, we speculate that this very similar pathophysiology we have observed in ePH may account for their adverse outcomes recently described by others.^15^ However, the present study was cross-sectional by design and future longitudinal studies are needed to address the underlying link between ePH and long-term outcomes.

ePH and rPH also had chronotropic incompetence that contributed to their reduced exercise capacity (Table 3). In ePH, this occurred largely in participants aged >50 years (Table S2) and was not strictly related to medication use (Table S3). In resting established PH, chronotropic incompetence has been associated with right heart failure and autonomic imbalance.^33–36^ Additionally, recent data suggest that in post-pulmonary endarterectomy patients with ePH, chronotropic incompetence might also occur.^37^ In our study, the chronotropic incompetence observed in older ePH patients was accompanied by indices of increased right ventricular work to an increased pulmonary vascular load (Table S3), suggesting therefore association with disease severity (i.e. autonomic imbalance due to right ventricular dysfunction^35,36^). rPH also had a reduced peripheral oxygen extraction, as seen by the blunted Ca-vO_2_ difference at peak exercise (Table 3), which likely reflect an associated skeletal muscle dysfunction^38,39^ and the multifactorial causes of exercise limitation in rPH.^40,41^

Taken together, the current findings provide important insight into the mechanisms of exercise intolerance in early pulmonary vascular disease. The results confirm previous non-invasive exercise findings in resting established PH^41^ and additionally suggest that oxygen delivery is impaired in the early stages of pulmonary vascular disease (i.e. ePH).

Recently, Kovacs et al. demonstrated that patients with borderline mPAP have decreased exercise capacity,^4^ a finding confirmed by the current study (Table 2). However, our data indicate that patients with borderline mPAP have a heterogeneous pulmonary vascular response to exercise, including patients with and without ePH, and that only those who develop ePH will suffer a functional limitation (Fig. 4). Therefore, our data suggest that ePH rather than borderline mPAP per se more precisely reflects an early pathophysiological stage of pulmonary vascular disease.

Assuming that pulmonary vascular dysfunction is a progressive phenomenon and that its late diagnosis is a major contributor to patient’s poor outcomes,^42^ considering ePH (rather than borderline resting mPAP) as an early stage of pulmonary vascular disease might be of major clinical relevance, potentially allowing early and more effective therapeutic interventions that could impact PH natural history.

### Predictive value of RHC for ePH

In addition to resting mPAP, other RHC measurements such as PVR and PVC also provided predictive information regarding ePH occurrence. This finding is of major clinical relevance and may provide additional framework for the identification of subgroups at a higher risk for ePH development and should stimulate further investigation. However, given the just moderate predictive value obtained from these variables in the current study (AUC = 0.76–0.78) and the substantial variability of pulmonary vascular responses across different resting RHC patterns, exercise hemodynamics should remain indispensable for the detection of early stages of pulmonary vascular dysfunction until more robust evidence is generated.

### Limitations

The study sample was derived from a tertiary dyspnea center and therefore the generalization of our findings should be done with caution. Non-PH patients were symptomatic and our sample reflect a heterogeneous population undergoing investigation for unexplained exertional intolerance. rPH and ePH patients also represent a heterogenous population that included patients with suspected PH of different causes. However, a subanalysis according to cardiovascular co-morbidities and PH risk factors revealed similar findings compared to those of the entire population (Tables S4 and S5), indicating that the mechanisms underlying their impaired exercise capacity are similar and that the hemodynamic profile had a central role influencing exercise intolerance. Our rPH population was relatively small in sample size due to the infrequency that participants with established resting disease undergo iCPET in our center and was older compared to non-PH and ePH, what might have influenced our rPH exercise hemodynamic findings. Additionally, due to the nature of the iCPET clinical referrals (i.e. investigation of unexplained exercise intolerance), rPH was mild in severity, which likely mitigated the changes during exercise that would have been seen with more severe disease. However, rPH were selected based on RHC criteria supported by current guidelines^22^ and represent a well established spectrum of pulmonary vascular disease.

For the ePH definition, we used age-related mPAP and PVR thresholds, derived from the study of physiologically normal participants that underwent an identical upright maximum incremental cycling exercise protocol to the one used in the current study.^18^ This age-specific ePH criteria addresses one of the major concerns that led to the exclusion of the ePH definition from the PH guidelines in 2008 (i.e. the uncertainty of the upper limits of normal for pulmonary hemodynamics in participants aged >50 years) and therefore likely decreases the number of false positive/negative diagnoses of ePH as a function of normal aging. The use of exercise hemodynamic thresholds determined by age is supported by additional recent evidence that pulmonary hemodynamics vary according to age in well-defined normal participants.^43^ Nonetheless, there was 92.66% concordance between the ePH definition used in the current study and the alternative proposed ePH criteria that uses a peak mPAP > 30 mmHg and peak TPR > 3 WU regardless of age.^13^

A subanalysis comparing the different ePH criteria is presented in the online supplementary material (Fig. S2 and Tables S6–S8). Considering peak VO_2_ as a marker of the presence/absence of disease, the age-specific ePH criteria demonstrated better sensitivity for participants aged ≤50 years and better specificity for participants aged >50 years (Table S4). These additional findings reinforce the potential role of age-specific exercise hemodynamic thresholds to identify clinically relevant abnormal pulmonary hemodynamic responses to exercise. However, our ePH criteria has not been externally validated and therefore further studies are necessary to confirm our findings.

Resting RHC CO was calculated using the Fick principle and an estimated VO_2_; therefore, resting supine CO-derived variables should be interpreted with caution. We did not use high-fidelity catheters to acquired pulmonary hemodynamic measurements, and more specifically pulmonary pulse pressure, during exercise; therefore, PVC findings should be carefully interpreted. During iCPET, pulmonary pressures were either measured through a passive exhalation,^23^ or using an electronic average over the respiratory cycle.^24^ The use of different techniques to measure pulmonary pressures might be considered a confounding factor, but it has been our observation that the pulmonary vascular pressures obtained during the passive exhalation technique do not differ from an average through the respiratory cycle in patients able to perform both. Finally, we did not directly measure right ventricular contractile reserve during exercise and did not evaluate the long-term prognostic implications of ePH. Therefore, future studies are needed to address these issues.

## Conclusions

The prevalence of ePH increases as a function of higher resting mPAP values currently considered in the normal range and is most frequently found in borderline mPAP. The development of ePH substantively affects exercise capacity regardless of the resting mPAP, and ePH and rPH share an underlying impaired oxygen delivery, increased right heart afterload, signs of right ventricular/pulmonary vascular uncoupling, and chronotropic incompetence that reduce exercise capacity. The current findings indicate that in the presence of borderline mPAP at RHC, evaluation of the pulmonary circulation under the stress of exercise is warranted to uncover physiologically and clinically relevant early pulmonary vascular disease.

## Supplementary Material

Supplementary material
