# Supplementary material for: Functional impact of exercise pulmonary hypertension in patients with borderline resting pulmonary arterial pressure
Source: Pulm Circ. 2017 Jun 8;7(3):654–65. doi: 10.1177/2045893217709025 (PMC5841910; doi:10.1177/2045893217709025)
Supplement: Supplementary material [file SupplementaryMaterial025.pdf]

SUPPLEMENTAL MATERIAL

Functional impact of exercise pulmonary hypertension in patients with borderline resting pulmonary arterial pressure

Figure S1. Distribution of resting mean pulmonary arterial pressure (mPAP) at right heart catheterization (RHC) for patients with mPAP <25 mmHg and PAWP ≤15 mmHg (n=312)

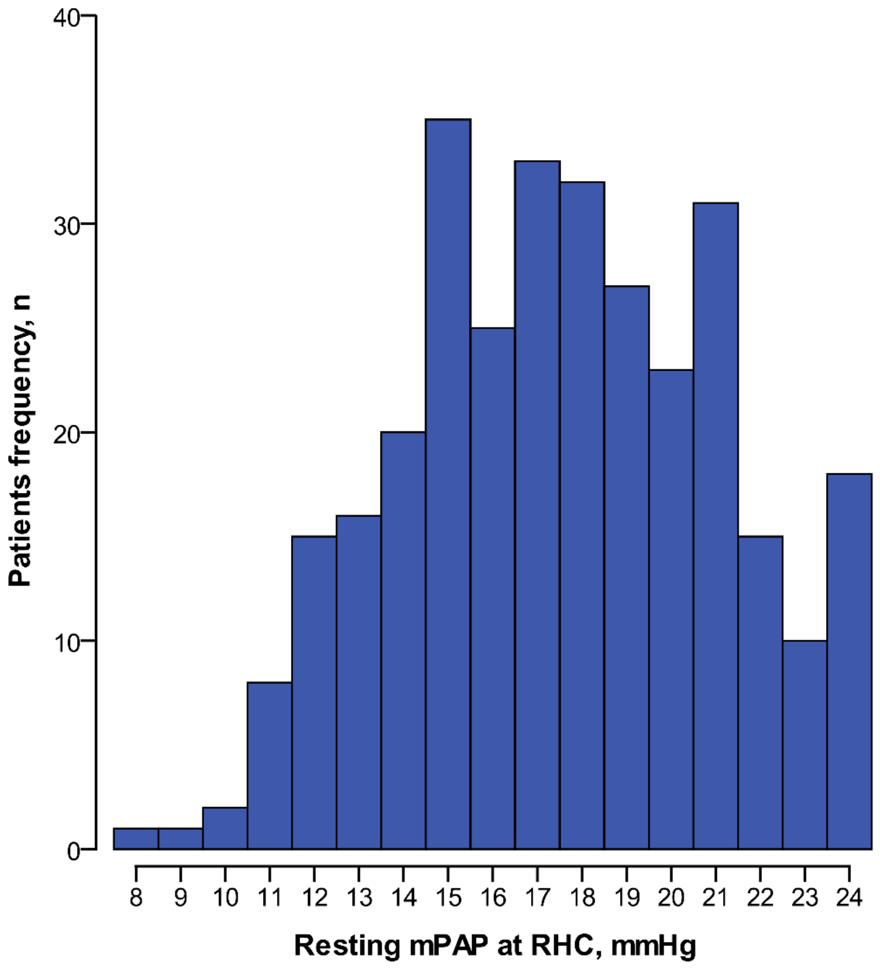

Table S1. Baseline characteristics and right heart catheterization data of patients without resting/exercise pulmonary hypertension (non-PH), with precapillary exercise hypertension (ePH) and with resting precapillary pulmonary hypertension (rPH)

	**non-PH**	**ePH**	**rPH**
**Subjects**	**224**	**35**	**16**
Age, years	53 ± 16	59 ± 16	62 ± 11
Females, n (%)	150 (67)	18 (51)	11 (69)
BMI, Kg/m^2	27.1 ± 5.9	30.4 ± 5.3 *	28.7 ± 5.5
Hemoglobin, g/dL	14.0 ± 1.5	13.9 ± 1.6	14.0 ± 1.7
**Comorbidities, n (%)**			
None	104 (46)	8 (23)	3 (19)
Hypertension	74 (33)	19 (54)	10 (63)
Connective tissue disease	19 (8)	6 (17)	3 (19)
Diabetes mellitus	11 (5)	6 (17)	5 (31)
Lung disease	5 (2)	6 (17)	3 (19)
Smokers	7 (3)	1 (3)	0
History of pulmonary embolism	16 (7)	1 (3)	2 (13)
**Medications, n (%)**			
Diuretics	28 (13)	7 (20)	5 (31)
ACE inhibitor or ARB	66 (29)	15 (43)	10 (63)
Beta-adrenergic receptor blocker	34 (16)	10 (29)	5 (31)
Calcium channel blocker	20 (9)	3 (9)	1 (6)
**Pulmonary function testing**			
FEV_1, % predicted	92 ± 17	76 ± 18 *	71 ± 19 *
FVC, % predicated	92 ± 18	79 ± 18 *	71 ± 18 *
FEV_1 / FVC, % predicted	100 ± 9	95 ± 13 *	100 ± 14

**Echocardiography**			
LA AP diameter, mm	36 ± 5	35 ± 6	33 ± 4
LVEF, %	62 ± 5	62 ± 5	63 ± 4
TRV, m/s	2.3 ± 0.3	2.7 ± 0.3 *	3.2 ± 0.7 * †
Estimated sPAP, mmHg	25 ± 8	31 ± 7 *	45 ± 16 * †
**Right heart catheterization**			
RA, mmHg	6 ± 2	7 ± 3	7 ± 3
mPAP, mmHg	17 ± 3	20 ± 3 *	36 ± 11 * †
PAWP, mmHg	10 ± 3	11 ± 3	10 ± 3
TPG, mmHg	7 ± 3	10 ± 3 *	27 ± 10 * †
CO, L/min	5.4 ± 1.1	4.9 ± 1.0 *	5.0 ± 1.0
CI, L/min/m^2	2.9 ± 0.5	2.6 ± 0.6 *	2.7 ± 0.4
TPR, WU	3.2 ± 0.8	4.3 ± 1.2 *	7.7 ± 3.1 * †
PVR, WU	1.3 ± 0.5	2.1 ± 0.8 *	5.7 ± 2.8 * †
PVC, ml/mmHg	6.2 ± 2.1	4.3 ± 1.6 *	2.2 ± 0.8 * †

Data are presented as n, n (%) or mean ± standard deviation. BMI: body mass index; ACE: angiotensin-converting- enzyme; ARB: angiotensin II receptor antagonist; FEV_1: forced expiratory volume in 1s; FVC: forced vital capacity, LA AP: left atrium antero-posterior; LVEF: left ventricular ejection fraction; TRV: tricuspid regurgitant jet velocity; sPAP: systolic pulmonary arterial pressure; RAP: right atrial pressure; mPAP: mean pulmonary arterial pressure; PAWP: pulmonary arterial wedge pressure; TPG: transpulmonary gradient; CO: cardiac output; CI: cardiac index; TPR: total pulmonary vascular resistance; PVR: pulmonary vascular resistance; PVC: pulmonary vascular compliance.

* $p < 0.05$ compared with non-PH

† $p < 0.05$ comparing rPH vs. ePH

Table S2. Functional and pathophysiological determinants of exercise pulmonary hypertension (ePH) during upright invasive cardiopulmonary exercise testing in subjects ≤50 years old vs. >50 years old

	**≤50 years**		**p value**	**>50 years**		**p value**
	**non-PH**	**ePH**		**non-PH**	**ePH**	
**Subjects**	90	15		134	20	
Age, years	37 ± 10	43 ± 7	0.046	64 ± 9	71 ± 8	<0.001
Female, n (%)	68 (76)	10 (67)	0.527	82 (61)	8 (40)	0.090
BMI, Kg/m2	26.1 ± 6.6	30.9 ± 5.0	<0.001	27.8 ± 5.3	30.0 ± 5.6	0.120
Beta-adrenergic receptor blocker	11 (12)	1 (7)	0.999	23 (17)	9 (45)	0.008
**Exercise capacity**						
Maximum Work rate, Watts	153 ± 48	127 ± 40	0.080	113 ± 43	78 ± 33	<0.001
Peak VO_2, % predicted	89 ± 19	72 ± 18	0.001	87 ± 18	64 ± 12	<0.001
Peak VO_2, ml/kg/min	25.2 ± 7.6	17.5 ± 4.4	<0.001	18.3 ± 5.4	12.5 ± 3.1	<0.001
VO_2 at AT, % VO_{2MAX} predicted	46 ± 10	43 ± 12	0.085	49 ± 12	38 ± 10	<0.001
**Determinants of exercise capacity**						
Peak CaO_2, mL/dL	19.4 ± 2.2	19.0 ± 1.8	0.439	19.2 ± 2.2	17.5 ± 2.6	0.003
Peak CvO_2, mL/dL	6.9 ± 1.9	7.1 ± 2.4	0.734	6.4 ± 1.6	5.5 ± 1.6	0.022
Peak $Ca\text{-}vO_2$, mL/dL	12.5 ± 2.0	11.9 ± 2.9	0.461	12.8 ± 1.8	12.0 ± 2.2	0.101

Peak CO, L/min	14.9 ± 4.0	12.7 ± 1.5	<0.001	11.3 ± 3.1	8.3 ± 1.5	<0.001
Peak CI, L/min/m^2	8.0 ± 1.8	6.5 ± 0.5	<0.001	5.9 ± 1.4	4.3 ± 0.7	<0.001
Peak SV, mL	93 ± 27	84 ± 14	0.236	84 ± 22	80 ± 13	0.531
Peak SVI, mL/m^2	49 ± 11	42 ± 5	0.009	44 ± 9	40 ± 7	0.175
Peak heart rate, bpm	164 ± 21	154 ± 16	0.026	136 ± 21	109 ± 19	<0.001
Peak heart rate, % predicted	89 ± 11	87 ± 9	0.241	87 ± 12	72 ± 12	<0.001
Peak DO_2, mL/min	2,908 ± 907	2,422 ± 408	0.079	2,197 ± 728	1,452 ± 309	<0.001
Peak DO_2, mL/kg/min	39.3 ± 11.2	28.1 ± 4.4	<0.001	27.5 ± 8.2	17.6 ± 4.1	<0.001
**Pulmonary pressures**						
Peak mPAP, mmHg	25 ± 6	38 ± 6	<0.001	27 ± 6	42 ± 8	<0.001
Peak PAWP, mmHg	11 ± 4	14 ± 4	0.017	11 ± 4	17 ± 6	<0.001
Peak TPG, mmHg	14 ± 5	24 ± 7	<0.001	16 ± 5	25 ± 7	<0.001
**Indices of right ventricular and pulmonary vascular load**						
Peak RAP, mmHg	5 ± 4	9 ± 4	<0.001	6 ± 3	10 ± 7	0.005
Peak TPR, WU	1.7 ± 0.4	3.0 ± 0.5	<0.001	2.5 ± 0.7	5.2 ± 1.5	<0.001
Peak PVR, WU	0.9 ± 0.3	1.9 ± 0.5	<0.001	1.5 ± 0.6	3.0 ± 0.8	<0.001
Peak PVC, mL/mmHg	3.9 ± 1.7	2.9 ± 1.2	0.042	3.2 ± 1.1	2.3 ± 1.2	<0.001
Peak RVSWI, g/m/m^2	17.5 ± 7.1	22.2 ± 5.1	0.004	16.5 ± 6.0	23.2 ± 5.9	<0.001

Data are presented as n or mean ± standard deviation. BMI: body mass index; VO_2: oxygen uptake; VO_{2MAX}: maximal oxygen uptake; AT: anaerobic threshold; CaO_2: arterial oxygen content; CvO_2: mixed-venous oxygen content; Ca-vO_2: arterial–mixed venous oxygen content

difference; CO: cardiac output; CI: cardiac index; SV: stroke volume; SVI: stroke volume index; DO_2: oxygen delivery; mPAP: mean pulmonary arterial pressure; PAWP: pulmonary arterial wedge pressure; TPG: transpulmonary gradient; RAP: right atrial pressure; TPR: total pulmonary vascular resistance; PVR: pulmonary vascular resistance; PVC: pulmonary vascular compliance; RVSWI: right ventricular stroke work index.

Table S3. Functional and pathophysiological determinants of exercise pulmonary hypertension (ePH) during upright invasive cardiopulmonary exercise testing in patients > 50 years old with and without beta-adrenergic receptor blocker use

	**ePH >50 years old**		**p-value**
	**Beta-blocker (-)**	**Beta-blocker (+)**	
**Subjects**	11	9	
Age, years	70 ± 9	73 ± 6	0.365
Female, n (%)	5 (45)	3 (33)	0.670
BMI, Kg/m2	28.0 ± 4.3	32.4 ± 6.3	0.080
**Exercise capacity**			
Maximum Work rate, Watts	84 ± 40	70 ± 20	0.360
Peak VO_2, % predicted	66 ± 12	62 ± 11	0.477
Peak VO_2, ml/kg/min	13.5 ± 3.4	11.3 ± 2.4	0.110
VO_2 at AT, % VO_{2MAX} predicted	41 ± 10	35 ± 10	0.199
**Determinants of exercise capacity**			
Peak CaO_2, mL/dL	17.1 ± 2.2	18.0 ± 3.1	0.442
Peak CvO_2, mL/dL	5.5 ± 1.5	5.5 ± 1.9	0.990
Peak Ca-vO_2, mL/dL	11.6 ± 2.5	12.5 ± 1.8	0.354
Peak CO, L/min	8.6 ± 1.5	7.9 ± 1.4	0.282
Peak CI, L/min/m^2	4.5 ± 0.6	4.1 ± 0.7	0.147
Peak SV, mL	80 ± 10	81 ± 17	0.868

Peak SVI, mL/m^2	39 ± 6	41 ± 7	0.496
Peak heart rate, bpm	117 ± 15	100 ± 19	0.041
Peak heart rate, % predicted	76 ± 10	68 ± 13	0.126
Peak DO_2, mL/min	1,482 ± 320	1,416 ± 310	0.648
Peak DO_2, mL/kg/min	18.6 ± 3.5	16.3 ± 4.7	0.213
**Pulmonary pressures**			
Peak mPAP, mmHg	42 ± 8	42 ± 7	0.915
Peak PAWP, mmHg	14 ± 5	20 ± 7	0.053
Peak TPG, mmHg	27 ± 8	22 ± 3	0.071
**Indices of right ventricular and pulmonary vascular load**			
Peak RAP, mmHg	7 ± 5	13 ± 7	0.029
Peak TPR, WU	5.0 ± 1.3	5.5 ± 1.6	0.428
Peak PVR, WU	3.0 ± 1.0	2.9 ± 0.5	0.645
Peak PVC, mL/mmHg	2.6 ± 1.5	2.0 ± 0.4	0.162
Peak RVSWI, g/m/$m/^2$	24.2 ± 6.7	21.9 ± 5.0	0.413

Data are presented as n or mean ± standard deviation. BMI: body mass index; VO_2: oxygen uptake; VO_{2MAX}: maximal oxygen uptake; AT: anaerobic threshold; CaO_2: arterial oxygen content; CvO_2: mixed-venous oxygen content; Ca-vO_2: arterial–mixed venous oxygen content difference; CO: cardiac output; CI: cardiac index; SV: stroke volume; SVI: stroke volume index; DO_2: oxygen delivery; mPAP: mean pulmonary arterial pressure; PAWP: pulmonary arterial wedge pressure; TPG: transpulmonary gradient; RAP: right atrial pressure; TPR: total pulmonary vascular resistance; PVR: pulmonary vascular resistance; PVC: pulmonary vascular compliance; RVSWI: right ventricular stroke work index.

Table S4. Functional and pathohistological determinants of exercise pulmonary arterial hypertension (ePH) and resting precapillary pulmonary hypertension (rPH) during upright invasive cardiopulmonary exercise testing for patients with cardiovascular risk factors (hypertension, diabetes and/or smoking history)

	**non-PH**	**ePH**	**rPH**
**Subjects**	**78**	**22**	**10**
**Exercise capacity**			
Maximum Work rate, Watts	116 ± 44	96 ± 48	55 ± 37 * †
Peak VO_2, % predicted	87 ± 16	65 ± 12 *	68 ± 19 *
Peak VO_2, ml/kg/min	18.0 ± 5.2	14.1 ± 4.5 *	11.9 ± 2.9 *
VO_2 at AT, % VO_{2MAX} predicted	48 ± 10	38 ± 9 *	42 ± 13 *
**Determinants of exercise capacity**			
Peak CaO_2, mL/dL	19.2 ± 2.3	18.3 ± 2.8	16.3 ± 2.0 *
Peak CvO_2, mL/dL	6.4 ± 1.7	5.7 ± 1.7	6.0 ± 2.0
Peak $Ca\text{-}vO_2$, mL/dL	12.8 ± 1.8	12.6 ± 2.3	10.4 ± 2.1 * †
Peak CO, L/min	11.9 ± 3.6	9.6 ± 2.6 *	8.9 ± 2.0 *
Peak CI, L/min/m^2	6.0 ± 1.5	4.9 ± 1.1 *	4.8 ± 1.0 *
Peak SV, mL	87 ± 22	83 ± 15	77 ± 19
Peak SVI, mL/m^2	44 ± 8	42 ± 6	42 ± 8
Peak heart rate, bpm	136 ± 23	116 ± 24 *	118 ± 17 *
Peak heart rate, % predicted	86 ± 11	75 ± 12 *	77 ± 11
Peak DO_2, mL/min	2,308 ± 830	1,780 ± 638 *	1,474 ± 412 *
Peak DO_2, mL/kg/min	27.2 ± 8.6	20.5 ± 5.8 *	18.8 ± 4.9 *
**Pulmonary pressures**			
Peak mPAP, mmHg	27 ± 6	41 ± 8 *	58 ± 7 * †
Peak PAWP, mmHg	11 ± 4	16 ± 6 *	16 ± 6 * †

Peak TPG, mmHg	16 ± 6	25 ± 6 *	41 ± 7 * †
**Indices of right ventricular and pulmonary vascular load**			
Peak RAP, mmHg	6 ± 3	10 ± 6 *	10 ± 4 *
Peak TPR, WU	2.4 ± 0.7	4.7 ± 1.7 *	6.8 ± 2.1 * †
Peak PVR, WU	1.4 ± 0.6	2.8 ± 0.9 *	4.9 ± 1.7 * †
Peak PVC, mL/mmHg	3.4 ± 1.4	2.3 ± 0.7 *	1.4 ± 0.6 *
Peak RVSWI, g/m/m^2	17.3 ± 6.2	23.8 ± 5.2 *	34.7 ± 6.4 * †

Data are presented as n or mean ± standard deviation. VO_2: oxygen uptake; VO_{2MAX}: maximal oxygen uptake; AT: anaerobic threshold; CaO_2: arterial oxygen content; CvO_2: mixed-venous oxygen content; Ca-vO_2: arterial–mixed venous oxygen content difference; CO: cardiac output; CI: cardiac index; SV: stroke volume; SVI: stroke volume index; DO_2: oxygen delivery; mPAP: mean pulmonary arterial pressure; PAWP: pulmonary arterial wedge pressure; TPG: transpulmonary gradient; RAP: right atrial pressure; TPR: total pulmonary vascular resistance; PVR: pulmonary vascular resistance; PVC: pulmonary vascular compliance; RVSWI: right ventricular stroke work index.

* $p < 0.05$ compared with non-PH

† $p < 0.05$ comparing rPH vs. ePH

Table S5. Functional and pathohistological determinants of exercise pulmonary arterial hypertension (ePH) and resting precapillary pulmonary hypertension (rPH) during upright invasive cardiopulmonary exercise testing for patients with PH risk factors (connective tissue disease, lung disease and/or history of pulmonary embolism)

	**non-PH**	**ePH**	**rPH**
**Subjects**	**36**	**10**	**7**
**Exercise capacity**			
Maximum Work rate, Watts	109 ± 40	96 ± 43	53 ± 32 *
Peak VO_2, % predicted	81 ± 18	67 ± 12	61 ± 19 *
Peak VO_2, ml/kg/min	17.6 ± 5.6	14.7 ± 4.4	11.1 ± 1.9 *
VO_2 at AT, % VO_{2MAX} predicted	45 ± 11	40 ± 9	37 ± 13
**Determinants of exercise capacity**			
Peak CaO_2, mL/dL	18.6 ± 2.2	17.9 ± 2.9	16.0 ± 1.0 *
Peak CvO_2, mL/dL	6.3 ± 1.4	6.3 ± 1.5	6.4 ± 1.5
Peak $Ca\text{-}vO_2$, mL/dL	12.3 ± 1.9	11.6 ± 2.4	9.5 ± 1.4 *
Peak CO, L/min	11.9 ± 3.8	9.6 ± 2.9	9.6 ± 3.0
Peak CI, L/min/m^2	6.1 ± 1.7	5.0 ± 1.2	5.0 ± 1.2
Peak SV, mL	84 ± 24	77 ± 12	76 ± 26
Peak SVI, mL/m^2	43 ± 10	38 ± 6	39 ± 10
Peak heart rate, bpm	141 ± 22	133 ± 25	129 ± 17
Peak heart rate, % predicted	86 ± 10	82 ± 11	82 ± 10
Peak DO_2, mL/min	2,196 ± 713	1,736 ± 700	1,532 ± 469 *
Peak DO_2, mL/kg/min	26.9 ± 9.1	21.4 ± 7.3	18.9 ± 4.0 *
**Pulmonary pressures**			
Peak mPAP, mmHg	27 ± 5	43 ± 9 *	56 ± 9 * †
Peak PAWP, mmHg	10 ± 4	15 ± 4 *	13 ± 4

Peak TPG, mmHg	17 ± 6	27 ± 9 *	43 ± 9 * †
**Indices of right ventricular and pulmonary vascular load**			
Peak RAP, mmHg	6 ± 4	9 ± 7	7 ± 3
Peak TPR, WU	2.4 ± 0.7	4.8 ± 1.8 *	6.4 ± 2.4 * †
Peak PVR, WU	1.5 ± 0.6	2.8 ± 1.1 *	5.0 ± 2.1 * †
Peak PVC, mL/mmHg	3.5 ± 1.4	2.1 ± 0.7 *	1.4 ± 0.7 *
Peak RVSWI, $g/m/m^2$	16.7 ± 7.1	22.6 ± 7.1 *	32.8 ± 5.6 * †

Data are presented as n or mean ± standard deviation. VO_2: oxygen uptake; VO_{2MAX}: maximal oxygen uptake; AT: anaerobic threshold; CaO_2: arterial oxygen content; CvO_2: mixed-venous oxygen content; Ca-vO_2: arterial–mixed venous oxygen content difference; CO: cardiac output; CI: cardiac index; SV: stroke volume; SVI: stroke volume index; DO_2: oxygen delivery; mPAP: mean pulmonary arterial pressure; PAWP: pulmonary arterial wedge pressure; TPG: transpulmonary gradient; RAP: right atrial pressure; TPR: total pulmonary vascular resistance; PVR: pulmonary vascular resistance; PVC: pulmonary vascular compliance; RVSWI: right ventricular stroke work index.

* $p < 0.05$ compared with non-PH

† $p < 0.05$ comparing rPH vs. ePH

Table S6: Exercise pulmonary arterial hypertension (ePH) diagnosis: age-specific peak mean pulmonary arterial pressure (mPAP) and peak pulmonary vascular resistance (PVR) criteria (see methods) vs. peak mPAP >30 mmHg and peak total pulmonary vascular resistance (TPR) >3 WU.

		**Peak mPAP >30 mmHg + Peak TPR >3WU**		
		**non-PH**	**ePH**	**Total**
**Age-specific**	**non-PH**	212	12 *	224
	**ePH**	7 †	28	35
	**Total**	219	40	259

* Mean age of 67±10 years (n=12/12 >50 years old) and peak VO_2 of 81±12% predicted.

† Mean age 40±7 years (n=7/7 ≤50 years old) and peak VO_2 was 69±20% predicted.

Please see figure S2 for individual pulmonary hemodynamic data.

Figure S2: (A) Peak mean pulmonary arterial pressure (mPAP) vs. peak cardiac output, and (B) Peak pulmonary vascular resistance (PVR) vs. peak total pulmonary vascular resistance (TPR) in 259 subjects with normal resting hemodynamics and without left heart disease during exercise (for detailed information please see methods and figure 1b).

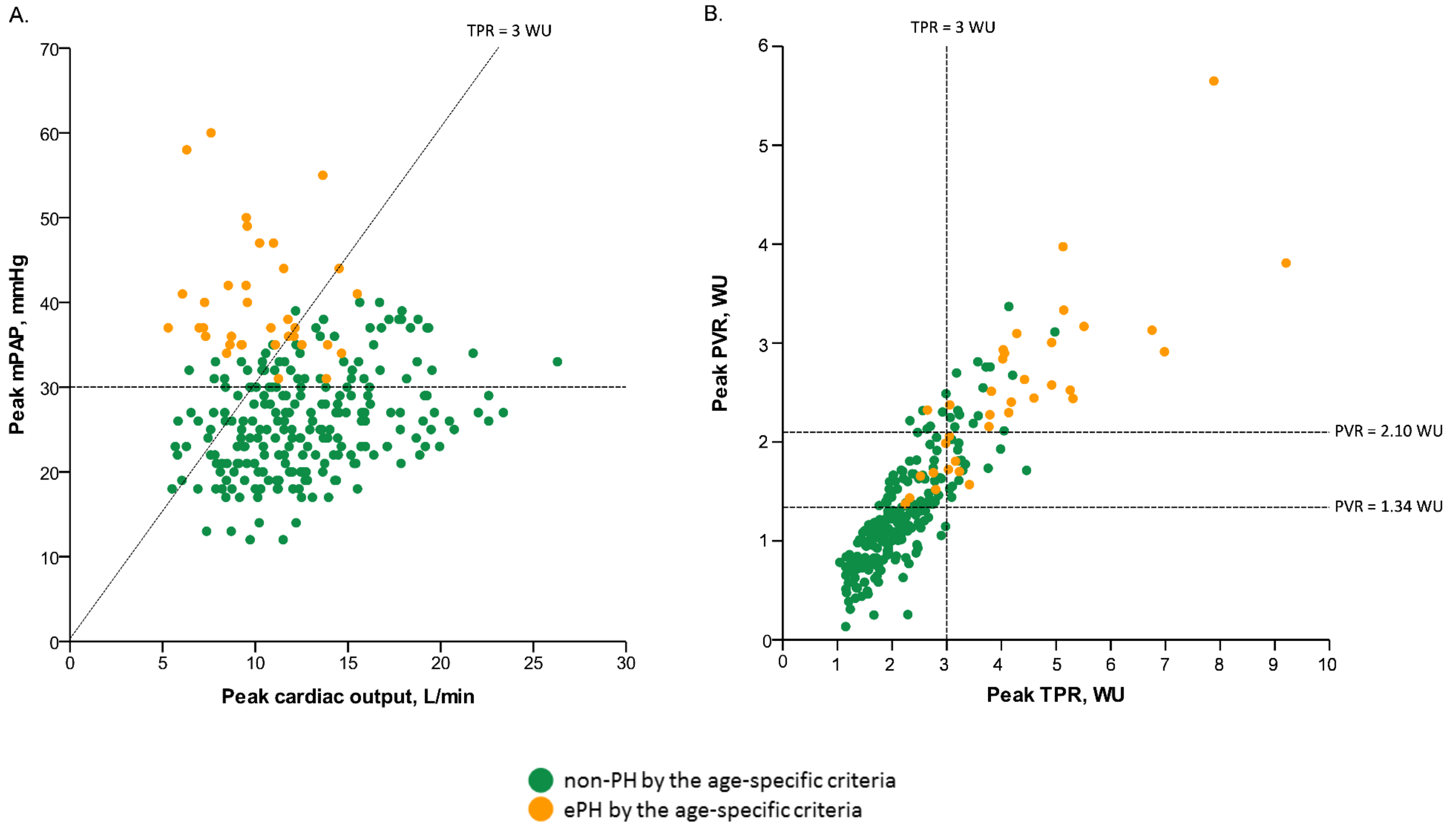

Table S7. Baseline characteristics and right heart catheterization data of exercise pulmonary hypertension (ePH) defined by a peak mean pulmonary arterial pressure >30 mmHg and a peak total pulmonary vascular resistance (TPR) >3 WU in relation to resting precapillary pulmonary hypertension (rPH) and patients without resting/exercise pulmonary hypertension (non-PH)

	**non-PH**	**ePH**	**rPH**
**Subjects**	219	40	**16**
Age, years	52 ± 16	65 ± 13 *	62 ± 11 *
Females, n (%)	146 (67)	22 (55)	11 (69)
BMI, Kg/m^2	27.2 ± 6.0	29.6 ± 5.0	28.7 ± 5.5
Hemoglobin, g/dL	14.0 ± 1.5	13.9 ± 1.4	14.0 ± 1.7
**Comorbidities, n (%)**			
None	106 (48)	6 (15)	3 (19)
Hypertension	69 (32)	24 (60)	10 (63)
Connective tissue disease	18 (8)	7 (18)	3 (19)
Diabetes mellitus	11 (5)	6 (15)	5 (31)
Lung disease	6 (3)	5 (13)	3 (19)
Smokers	6 (3)	2 (5)	0
History of pulmonary embolism	14 (6)	3 (8)	2 (13)
**Pulmonary function testing**			
FEV_1, % predicted	92 ± 17	78 ± 19 *	71 ± 19 *
FVC, % predicated	92 ± 17	80 ± 18 *	71 ± 18 *
FEV_1 / FVC, % predicted	99 ± 9	98 ± 12	100 ± 14
**Echocardiography**			
LA AP diameter, mm	36 ± 5	36 ± 6	33 ± 4
LVEF, %	62 ± 4	62 ± 5	63 ± 4

TRV, m/s	2.4 ± 0.3	2.5 ± 0.4	3.2 ± 0.7 * †
Estimated sPAP, mmHg	25 ± 8	27 ± 8	45 ± 16 * †
**Right heart catheterization**			
RA, mmHg	6 ± 2	6 ± 3	7 ± 3
mPAP, mmHg	17 ± 3	21 ± 3 *	36 ± 11 * †
PAWP, mmHg	10 ± 3	11 ± 3	10 ± 3
TPG, mmHg	7 ± 2	10 ± 3 *	27 ± 10 * †
CO, L/min	5.4 ± 1.1	5.0 ± 1.1 *	5.0 ± 1.0
CI, L/min/m^2	2.9 ± 0.5	2.6 ± 0.5 *	2.7 ± 0.4
TPR, WU	3.2 ± 0.8	4.4 ± 1.1 *	7.7 ± 3.1 * †
PVR, WU	1.2 ± 0.5	2.1 ± 0.8 *	5.7 ± 2.8 * †
PVC, ml/mmHg	6.2 ± 2.1	4.3 ± 1.6 *	2.2 ± 0.8 * †

Data are presented as n, n (%) or mean ± standard deviation. BMI: body mass index; FEV_1: forced expiratory volume in 1s; FVC: forced vital capacity, LA AP: left atrium antero-posterior; LVEF: left ventricular ejection fraction; TRV: tricuspid regurgitant jet velocity; sPAP: systolic pulmonary arterial pressure; RAP: right atrial pressure; mPAP: mean pulmonary arterial pressure; PAWP: pulmonary arterial wedge pressure; TPG: transpulmonary gradient; CO: cardiac output; CI: cardiac index; TPR: total pulmonary vascular resistance; PVR: pulmonary vascular resistance; PVC: pulmonary vascular compliance.

* p <0.05 compared with non-PH

† p <0.05 comparing rPH vs. ePH

Table S8. Functional and pathophysiological determinants of exercise pulmonary hypertension (ePH) defined by a peak mean pulmonary arterial pressure >30 mmHg and a peak total pulmonary vascular resistance (TPR) >3 WU in relation to resting precapillary pulmonary hypertension (rPH) and patients without resting/exercise pulmonary hypertension (non-PH) during upright invasive cardiopulmonary exercise testing

	**non-PH**	**ePH**	**rPH**
**Subjects**	219	40	16
**Exercise capacity**			
Maximum Work rate, Watts	131 ± 49	92 ± 35 *	77 ± 49 *
Peak VO_2, % predicted	87 ± 19	71 ± 14 *	68 ± 17 *
Peak VO_2, ml/kg/min	21.3 ± 7.2	14.3 ± 3.7 *	14.3 ± 5.8 *
VO_2 at AT, % VO_{2MAX} predicted	48 ± 12	42 ± 11 *	39 ± 11 *
**Determinants of exercise capacity**			
Peak CaO_2, mL/dL	19.3 ± 2.2	18.3 ± 2.3 *	16.7 ± 2.0 * †
Peak CvO_2, mL/dL	6.6 ± 1.8	6.0 ± 1.7	6.3 ± 1.8
Peak $Ca\text{-}vO_2$, mL/dL	12.6 ± 2.0	12.2 ± 2.1	10.4 ± 1.8 * †
Peak CO, L/min	13.0 ± 3.8	9.4 ± 2.1 *	10.3 ± 3.4 *
Peak CI, L/min/m^2	6.8 ± 1.8	4.9 ± 1.0 *	5.6 ± 1.7 *
Peak SV, mL	88 ± 24	78 ± 13 *	79 ± 22
Peak SVI, mL/m^2	46 ± 10	40 ± 6 *	43 ± 10
Peak heart rate, bpm	148 ± 25	125 ± 26 *	131 ± 23 *
Peak heart rate, % predicted	88 ± 11	80 ± 14 *	82 ± 11

Peak DO_2, mL/min	2,521 ± 869	1,731 ± 472 *	1,756 ± 720 *
Peak DO_2, mL/kg/min	32.7 ± 11.0	21.2 ± 6.1 *	23.1 ± 10.2 *
**Pulmonary pressures**			
Peak mPAP, mmHg	26 ± 6	39 ± 7 *	58 ± 11 * †
Peak PAWP, mmHg	11 ± 4	15 ± 5 *	15 ± 7 *
Peak TPG, mmHg	15 ± 5	24 ± 6 *	43 ± 10 * †
**Indices of right ventricular and pulmonary vascular load**			
Peak RAP, mmHg	5 ± 4	9 ± 5 *	9 ± 5 *
Peak TPR, WU	2.1 ± 0.6	4.4 ± 1.4 *	6.1 ± 2.3 * †
Peak PVR, WU	1.2 ± 0.5	2.6 ± 0.8 *	4.6 ± 1.8 * †
Peak PVC, mL/mmHg	3.5 ± 1.4	2.4 ± 1.0 *	1.6 ± 0.7 *
Peak RVSWI, $g/m/m^2$	17.0 ± 6.5	21.7 ± 5.6 *	36.7 ± 10.9 * †

Data are presented as n or mean ± standard deviation. VO_2: oxygen uptake; VO_{2MAX}: maximal oxygen uptake; AT: anaerobic threshold; CaO_2: arterial oxygen content; CvO_2: mixed-venous oxygen content; Ca-vO_2: arterial–mixed venous oxygen content difference; CO: cardiac output; CI: cardiac index; SV: stroke volume; SVI: stroke volume index; DO_2: oxygen delivery; mPAP: mean pulmonary arterial pressure; PAWP: pulmonary arterial wedge pressure; TPG: transpulmonary gradient; RAP: right atrial pressure; TPR: total pulmonary vascular resistance; PVR: pulmonary vascular resistance; PVC: pulmonary vascular compliance; RVSWI: right ventricular stroke work index.

* $p < 0.05$ compared with non-PH; † $p < 0.05$ comparing rPH vs. ePH
